# Genomic epidemiology of the opportunistic pathogen *Staphylococcus coagulans* from companion dogs

**DOI:** 10.1099/jmm.0.001407

**Published:** 2021-08-25

**Authors:** Gavin K. Paterson

**Affiliations:** ^1^​ Royal Dick School of Veterinary Studies and The Roslin Institute, University of Edinburgh, Edinburgh EH25 9RG, UK

**Keywords:** coagulase-positive staphylococci, methicillin resistance, *Staphylococcus coagulans*, veterinary microbiology

## Abstract

**Introduction:**

*
Staphylococcus coagulans* (formerly *Staphylococcus schleiferi* subsp. *coagulans *) is a common commensal and opportunistic pathogen of companion dogs. It carries a range of antimicrobial resistance genes and is an occasional zoonotic pathogen.

**Hypothesis/Gap Statement:**

Despite the potential insight offered by genome sequencing into the biology of *S. coagulans *, few genomes are currently available for study.

**Aim:**

To sequence and analyse *S. coagulans* genomes to improve understanding of this organism’s molecular epidemiology, antimicrobial resistance and bacterium–host interactions.

**Methodology:**

Twenty-five genomes of clinical isolates collected at a veterinary referral hospital in Scotland, UK, were sequenced with Illumina technology. These genomes were analysed by a series of bioinformatics tools along with 16 previously sequenced genomes.

**Results:**

Phylogenetic comparison of the 41 genomes shows that the current *S. coagulans* phylogeny is dominated by clades of closely related isolates, at least one of which has spread internationally. Ten of the 11 methicillin-resistant *S. coagulans* genomes in this collection of 41 encoded the *mecA* promoter and gene mutations that are predicted to render the isolates susceptible to penicillins in the presence of clavulanic acid, a feature only described to date in methicillin-resistant *Staphylococcus aureus *. Seven such isolates were from the current study and, in line with the genome-based prediction, all were susceptible to amoxicillin/clavulanic acid *in vitro. S. coagulans* shared very few highly conserved virulence-associated genes with *Staphylococcus pseudintermedius *, another common commensal and opportunistic canine pathogen.

**Conclusion:**

The availability of a further 25 genome sequences from clinical *S. coagulans* isolates will aid in better understanding the epidemiology, bacterial–host interactions and antimicrobial resistance of this opportunistic pathogen.

## Introduction

*Staphylococcus coagulans* was originally described in 1990 as *Staphylococcus schleiferi* subsp. *coagulans* [1] before being promoted, on the basis of genomic parameters, to a separate species in 2020 [2]. *S. coagulans* is primarily a commensal and opportunistic pathogen of companion dogs. It is frequently isolated from the skin [3] and the external ear canal [4] of healthy dogs as well as being associated with external ear otitis [1, 3–5] and pyoderma [4, 6–8]. While rare, there are also reports of *S. coagulans* causing opportunistic infections in compromised humans [9–13]. In addition to companion dogs and humans, the list of currently reported hosts or sites of *S. coagulans* isolation, to the best of my knowledge, is as follows; domestic cats [14], chicken meat [15], ready to eat retail fish [16], healthy feral and domestic pigeons [17], Adélie penguin (*Pygoscelis adeliae*) [18], South polar skua (*Stercorarius maccormicki*) [18], Weddell seal (*Leptonychotes weddellii*) [18], southern elephant seal (*Mirounga leonina*) [18, 19], grey seal (*Halichoerus grypus*) [19] and Antarctic fur seal (*Arctocephalus gazella*) [19]. Akin to many staphylococcal species, *S. coagulans* is therefore widely distributed in avian and mammalian host species and is probably found more widely among such host species than is presently documented.

As with other staphylococci [20], methicillin resistance is encoded by *mecA* in *S. coagulans* [8, 21, 22] and resistance against a range of other antimicrobials has been reported including penicillin [23, 24], erythromycin [8, 23–26], clindamycin [7, 8, 23–25], lincomycin [7], gentamicin [8, 23, 25, 26], fusidic acid [24], tetracycline [23, 25] and fluoroquinolones [7, 8, 24–26]. Not only could this resistance impede the successful treatment of *S. coagulans* infections, antimicrobial resistance genes have been shown to move between staphylococcal species and there is potential for *S. coagulans* to act as a genetic reservoir for the onward dissemination of resistance determinants to other staphylococci, including more pathogenic species such as *Staphylococcus aureus* and *Staphylococcus pseudintermedius* [27–30].

Despite the frequency of *S. coagulans* as an opportunistic pathogen in companion dogs and its zoonotic potential, relatively few genome sequences are available with which to inform our understanding of *S. coagulans* biology, such as its epidemiology, antimicrobial resistance and bacterium–host interactions. Ultimately, such data may facilitate new interventions to prevent, diagnose and treat *S. coagulans* infections. Reported herein is the genome sequencing and analysis of 25 *S. coagulans* clinical isolates collected at the Royal (Dick) School of Veterinary Studies Hospital for Small Animals, Scotland, UK. This collection comprises 24 canine isolates, of which seven are methicillin-resistant, and a single methicillin-sensitive feline isolate. The isolates are placed into wider context by phylogenetic comparison with other available genomes, resulting in a final collection of 41 genome-sequenced *S. coagulans* isolates.

## Methods

### Bacterial isolation, identification and antimicrobial sensitivity testing

Twenty-five *S. coagulans* study isolates were collected during routine diagnostic work performed at Easter Bush Pathology, Royal (Dick) School of Veterinary Studies [R(D)SVS], University of Edinburgh, from samples received from the R(D)SVS Hospital for Small Animals between 1 June 2017 and 31 August 2019. With the exception of a single isolate which failed genome sequencing, all isolates recovered during this time period are included in this study.

Clinical isolates were isolated on Columbia agar supplemented with 5 % horse blood (E and O Laboratory) and incubated atmospherically at 37 °C for 18–24 h. Isolates from samples screening for methicillin-resistant staphylococci were isolated on MRSA Brilliance (Oxoid) and incubated atmospherically at 37 °C for 24 h.

Isolates were identified and antimicrobial sensitivity testing was performed using a Vitek2 (bioMéurieux) following the manufacturer’s instructions. Using AST-GP80 cards, the following antimicrobials were tested: amoxicillin/clavulanic acid, benzylpenicillin, cefovecin, cefoxitin (screen), ceftiofur, chloramphenicol, clindamycin, doxycycline enrofloxacin, erythromycin, gentamicin, clindamycin (inducible resistance), kanamycin, marbofloxacin, neomycin, nitrofurantoin, oxacillin, pradofloxacin, tetracycline and trimethoprim/sulfamethoxazole. Interpretation was made according to the Clinical and Laboratory Standards Institute (CLSI) criteria (2017).

### Whole genome sequencing

Whole genome sequencing was performed by Microbes NG (University of Birmingham, UK) as described previously [19]. In brief, genomic DNA was extracted using Solid Phase Reversible Immobilization beads and genomic DNA libraries were prepared using the Nextera XT Library Prep Kit (Illumina) following the manufacturer’s protocol with the following modifications: input DNA is increased 2-fold, and PCR elongation time is increased to 45 s. Libraries were sequenced using Illumina sequencers (HiSeq/NovaSeq) using a 250 bp paired-end protocol. Reads were trimmed using Trimmomatic version 0.30 [31], using a sliding window quality cut-off of 15. Genome assembly was done *de novo* using SPAdes, version 3.7, with default parameters for 250 bp Illumina reads [32] and annotated by the NCBI Prokaryotic Genome Annotation Pipeline [33].

### Genome analysis

Study isolates were confirmed to belong to *S. coagulans* using the Type Strain Genome Server [34]. Acquired resistance genes were identified using ResFinder-4.1 employing the threshold of 80 % for percentage identity and minimum length of 80 % [35]. Virulence-factor genes were identified by blast using MyDbFinder 2.0 (https://cge.cbs.dtu.dk/services/MyDbFinder/) and a published list of *S. pseudintermedius* virulence-related gene sequences [36]. Thresholds of 90 % for percentage identity and 80 % for minimum length were applied to the virulence-related gene blast search. SCC*mec* typing from genome sequences was performed using SCC*mec*Finder [37].

Phylogenetic relationships between study isolates and previously sequenced, assembled and annotated *S. schleiferi* isolates [19, 38–41] were inferred using CSI Phylogeny 1.4 (Call SNPs and Infer Phylogeny) [42] using the type strain *S. coagulans* DSM 6628^T^ (GCA_002901995.1) as the reference genome and applying default settings [minimum depth at single nucleotide polymorphism (SNP) positions: 10×; minimum relative depth at SNP positions: 10 %, minimum distance between SNPs (prune): 10 bp; minimum SNP quality: 30; minimum read mapping quality: 25 and minimum Z-score: 1.96]. In total, 2 096 299 positions were found in all analysed genomes. *Staphylococcus schleiferi* ATCC 43808^T^ (GCA_011137195) was included as the outgroup to root the tree. The resultant tree was annotated using the Interactive Tree of Life (iTOL) [43].

### Data availability

Isolate metadata and nucleotide accessions are provided in Table S1 (available in the online version of this article).

## Results and discussion

### Isolate collection and phylogenetic analysis

Twenty-five *S. coagulans* isolates were collected and genome-sequenced during the collection period. Isolates were initially identified phenotypically as *S. schleiferi* by the Vitek2 during routine diagnostic work and subsequently shown to represent *S. coagulans* following genome sequencing and analysis using the Type Strain Genome Server. At the time of isolate collection, the Vitek2 platform was validated to identify *S. schleiferi* but was not able to differentiate the two subspecies *S. schleiferi* subsp. *schleiferi* and *S. schleiferi* subsp. *coagulans *; the latter was subsequently changed to a species in its own right, *S. coagulans,* in 2020 [2]. Hence, study isolates were originally identified as being *S. schleiferi* on the basis of phenotype and then identified definitively following genome sequencing. The Type Strain Genome Server uses Genome Blast Distance Phylogeny to delimitate bacterial species and was selected for the genome-based identification due to its comprehensive and curataed database of type strains. The 25 genomes sequenced in this study ranged in size from 2 426 360 to 2 544 913 bp with a mean of 2 483 639 bp. The G+C content ranged from 35.75 to 36.32 mol% with a mean of 35.91 mol%. These figures are comparable to those for the species type strain, DSM 6628^T^, which has a 2 443 567 bp genome with a G+C content of 35.83 mol%. A single isolate came from a cat with the remainder being from dogs. The most common sites of isolation were skin/wound/lesion (11 isolates) followed by ear swabs (six isolates; Table S1). Thirteen of the 25 isolates were susceptible to all 21 antimicrobials tested (Table S1). Seven isolates of these 25 were phenotyically methicillin-resistant, all of these seven were resistant to oxacillin but only two of them were resistant in cefoxitin. This agrees with previous data demonstrating that oxacillin is more reliable than cefoxitin for the detection of *mecA*-mediated methicillin resistance in *S. coagulans* [21]. All phenotypcially methicillin-resistant isolates encoded *mecA* on an SCC*mec* type V element except for 5909-02 which encoded *mecA* within an SCC*mec* type IVa element.

**Fig. 1.**
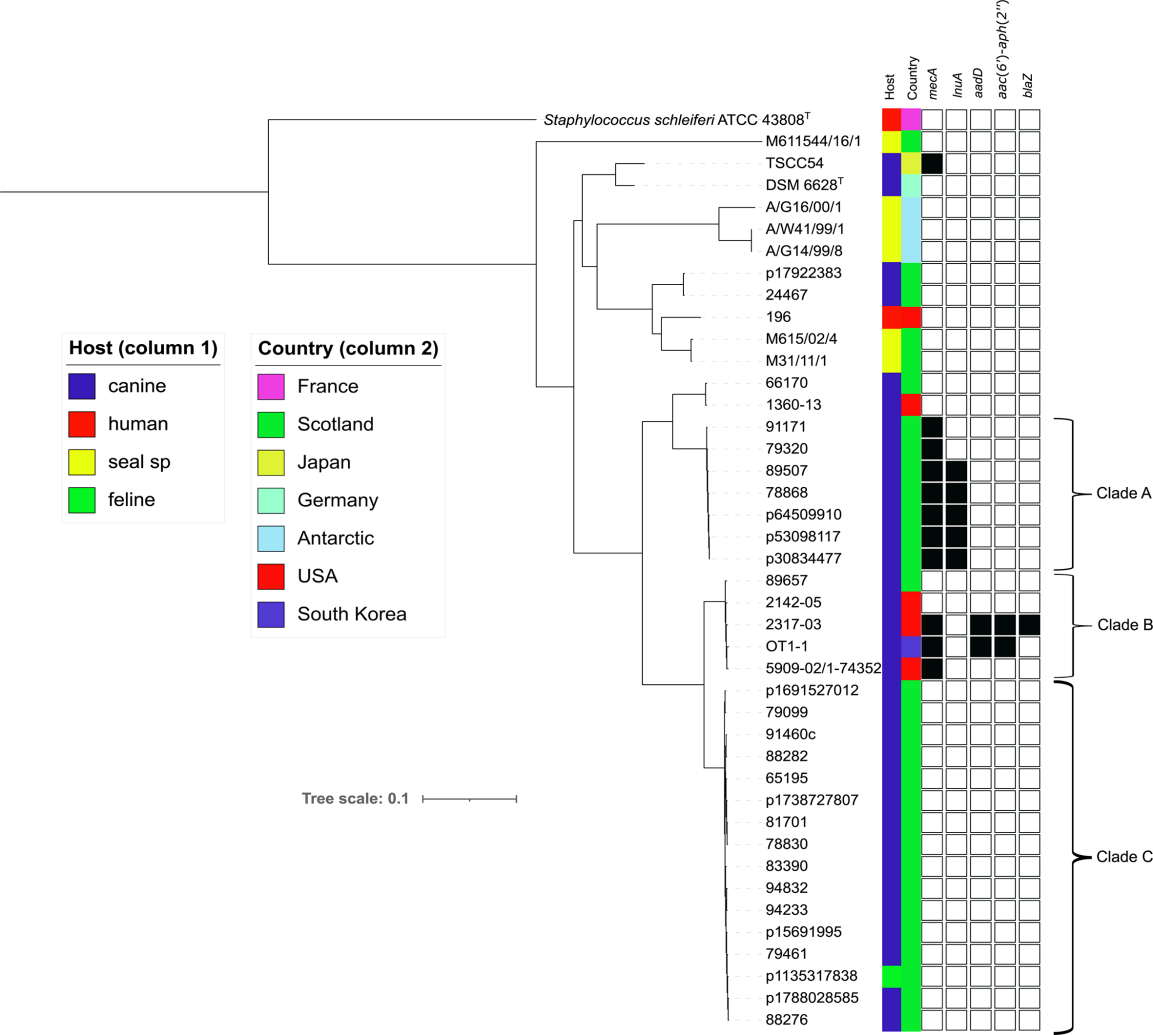
Phylogenetic analysis of currently available *S. coagulans* genome-sequenced isolates. The phylogeny was generated from SNPs across 2 096 299 positions in the core genome using CSI Phylogeny 1.4 [42] with *S. coagulans* DSM 6628^T^ as the reference genome and *S. schleiferi* ATCC 43808^T^ as the outgroup to root the tree. Host (column 1) and country of origin (column 2) are indicated in coloured columns. Presence (black square) or absence (white square) of indicated antimicrobial resistance genes are shown in subsequent columns. Genome accessions are provided in Table S1.

The 25 study isolates were compared with the 16 currently available *S. coagulans* genome-sequenced isolates (Table S1) using an SNP-based phylogenetic analysis. This phylogeny, comprising 41 isolates, shows that the currently sequenced *S. coagulans* population is dominated by three highly clonal clades, A–C, which together comprise 29 (71 %) of the available genome-sequenced isolates (Fig. 1). Clade A comprises of seven Scottish methicillin-resistant isolates with isolates separated by a pairwise average of 29 SNPs. Two isolates in this clade came from the same individual dog 54 days apart. These two isolates are separated by only two SNPs and indicate that colonization with the same strain of methicillin-resistant *S. coagulans* can last for at least 7 weeks. Clade B comprises only five isolates but includes isolates from Scotland, USA and South Korea separated by an average pairwise difference of 232 SNPs. This demonstrates the international dissemination of this lineage, which is further noteworthy with regard to variation in methicillin resistance among these isolates. Two of these isolates are methicillin-sensitive, and while three isolates are methicillin-resistant, two, 2317-03 and OT1-1, encode SCC*mec* type V with 5909-02 encoding SCC*mec* type IVa. Clade C is the largest cluster of related isolates, comprising 16 isolates, all from Scotland, and separated by an average pairwise difference of 90 SNPs. Among the canine isolates in Clade C is a single feline isolate, separated from the nearest canine isolate by 35 SNPs and thus demonstrating that different companion animal species can be infected by closely related *S. coagulans* strains.

Eleven isolates in the sequenced collection of 41 encoded *mecA* and in each case this was within an SCC*mec* type V apart from the aforementioned exception of type IVa encoded in isolate 5909-02. Other resistance genes were not common (Fig. 1). The lincomycin resistance gene *lnu*(A) [44] was present in five of the seven Scottish methicllin-resistant *S. coagulans* (MRSC) isolates in Clade A. The aminoglycoside resistance determinants, *aadD* and *acc*(6′)-*aph*(*2*′), were present in two previously sequenced isolates from South Korea and USA, OT1-1 and 2317-03, with *blaZ* also present in the latter isolate (Fig. 1).

### Suscpetibilty to amoxicillin/clavulanic acid

In addition to oxacillin, all seven Scottish MRSC isolates were resistant to the tested β-lactams penicillin, cefalotin and ceftiofur (Table S1). However, all seven were unexpectedly susceptible to amoxicillin/clavulanic acid. Similar unexpected susceptibility to penicillins and β-lactamase inhibitors has been reported in methicillin-resistant *S. aureus* (MRSA) [45]. In MRSA this susceptibility is conferred by a combination of two mutations in the *mecA* promoter region which lowers expression of the *mecA* gene and penicillin-binding protein 2a (PBP2a), and two substitutions in PBP2a (E246G or M122I) which increase its affinity for penicillins in the presence of clavulanic acid [45]. All seven Scottish MRSC isolates encoded the E246G mutation in PBP2a and the *mecA* promoter mutation *mecA*[−33]:C-T which, based on their characterization in MRSA, most probably confer their susceptibility to amoxicillin/clavulanic acid. To the best of my knowledge, this is the first demonstration of these *mecA* mutations and their association with amoxicillin/clavulanic acid susceptibility in methicillin-resistant staphylococci other than *S. aureus *. While not available for phenotypic testing, the previously sequenced MRSC isolates OT1-1 and TSCC54 possessed these same two mutations, suggesting that they too would be susceptible to amoxicillin/clavulanic acid. MRSC isolate 5909-02 is also likely to be susceptible, as it carried the E246G mutation in PBP2a and the second characterized *mecA* promoter mutation, *mecA*[−7]:G-T, associated with amoxicillin/clavulanic acid in MRSA. However, isolate 2317-03 demonstrates that these mutations are not ubiquitous in MRSC, as it lacked any of the four mutations and is therefore predicted to be resistant to amoxicillin/clavulanic acid.

Eight of the 25 study isolates displayed resistance to fluoroquinolones (Table S1). All seven MRSC isolates were resistant to enrofloxacin and marbofloxacin and intermediate with regard to pradofloxacin. A single methicillin-sensitive isolate, p17922383, showed resistance to all three tested fluoroquinolones. In staphylococci, four SNPs in *grlA* (G239T and G250A) and *gyrA* (C251T and A263G) are the mutations most commonly associated with fluoroquinolone resistance [46, 47]. All eight fluoroquinolone-resistant isolates carried the *gyrA* C251T mutation, which is probably responsible for this resistance.

### Virulence factors in *S. coagulans*

The virulence-associated gene reportiore of *S. coaglans* is, as yet, poorly characterized. Therefore all 41 *S. coagulans* genomes were investigated for the presence of 69 virulence-associated genes from another staphylococcal opportunistic pathogen of dogs, *S. pseudintermedius *, with similar epidemiology. Thresholds for nuclelotide identity and length match were set at 90 and 80% respectively. Thirty-nine isolates had no matches to any of the 69 virulence-associated genes. Staphylococcal entertoxin C3 (*sec3*) and toxic shock syndrome toxin (*tst*) were present in the two indistinguishable seal isolates, A/G14/99/8 and A/W41/99/1. The only other virulence-related gene found in this analsyis was the gene encoding the bacterocin and immunomodulatory peptide, BacSp222 [48], which is present in another seal isolate, M615/02/4. To the best of my knowledge, these represent the first reports of these virulence-associated genes in *S. coagulans *. These data indicate a limited sharing of highly conserved virulence-related genes between *S. coagulans* and *S. pseudintermedius *. Notably, no *S. coagulans* isolate carried *coa*, encoding staphylocoagulase, purported to be responsible for coagulase activity in staphylococci. The molecular basis for this activity in *S. coagulans* therefore remains to be defined. The availablity of an expanded collection of genome-sequenced *S. coagulans* will facilitate the future exploration of virulence-related genes and the bacterial–host interactions of this organism.

## Conclusion

This report describes the genome sequencing of 25 clinical isolates of S. *coagulans* from Scotland, UK, and their analysis with the 16 other available *S. coagulans* genomes. The resultant *S. coagulans* phylogeny is dominated by clusters of highly related isolates indicative of the clonal expansion of successful lineages, including their international dissemination. Many isolates are susceptible to all tested antimicrobials and lack antimcroibial resistance determinants. Few conserved virulence-related genes are shared with *S. pseudintermedius *, highlighting that much remains to be elucidated with regard to *S. coagulans* bacterial–host interactions.

## Supplementary Data

Supplementary material 2
